# The usefulness of dual mobility cups in primary total hip arthroplasty patients at a risk of dislocation

**DOI:** 10.1038/s41598-022-04774-2

**Published:** 2022-01-14

**Authors:** Nam Hoon Moon, Min Uk Do, Jung Shin Kim, Jae Seung Seo, Won Chul Shin

**Affiliations:** 1 Department of Orthopaedic Surgery, Pusan National University Hospital, Busan, Republic of Korea; 2 Department of Orthopaedic Surgery, Research Institute for Convergence of Biomedical Science and Technology, Pusan National University Yangsan Hospital, Pusan National University School of Medicine, 20 Geumo-ro, Mulgeum-eup, Yangsan, Gyeongsangnam-do 626-770 Republic of Korea

**Keywords:** Biotechnology, Diseases, Health care, Medical research, Signs and symptoms

## Abstract

This study aimed to evaluate the early results of primary total hip arthroplasty (THA) using dual mobility (DM) cups in patients at a risk of dislocation and compare them with that of fixed bearing (FB) THA. This retrospective study included patients who had undergone primary THA between January 2016 and December 2018 and were at a risk of dislocation. A propensity score-matched analysis was conducted for 63 THA procedures with vitamin-E infused highly cross-linked polyethylene (VEPE) DM bearing and 63 THA procedures performed with FB from the same manufacturer for a mean follow-up period of 3.1 and 3.5 years, respectively. The radiologic outcomes at the last follow-up and incidence of postoperative complications were evaluated and compared statistically between the two groups. The modified Harris hip score (mHHS) was used to assess patient-reported outcomes. Postoperative dislocation occurred in 4 cases (6.3%) in the FB group, but did not occur in the DM group (*p* = 0.042). There was no difference in the radiologic outcomes and postoperative complications between the two groups. The mHHS at the last follow-up showed satisfactory outcomes in both the groups (DM group, 90.5; FB group, 88.1), without a statistical difference between the groups. The early results of THA using VEPE DM bearing showed better outcomes than that of THA with FB for patients at a risk of dislocation. A longer follow-up period is recommended to assess the stability and overall outcomes.

## Introduction

Although total hip arthroplasty (THA) is one of the most successful surgical procedures developed in the twenty-first century, there is a lethal risk associated with this procedure. Among the complications that can occur after THA, instability is the second most common cause for revision surgery and is known to threaten the long-term success of THA^1^. The cause of prosthesis instability is multifactorial, and various efforts have been made to prevent dislocation after THA. Surgical techniques, such as the selection of surgical approaches, that can be controlled by an operator do not have a significant impact on development of the technique that can prevent dislocation. However, patient factors, such as neuromuscular disorder, abductor insufficiency, dysplastic hip, spinopelvic impairment, and previous surgical history, which cannot be controlled by an operator, are significant risk factors for dislocation after THA. Many techniques have been developed to control the factors affecting early dislocation. Large prosthetic heads, trochanteric advancement, constrained liners, modular components, and constrained and unconstrained dual mobility (DM) components are the different modalities used to decrease the incidence of dislocation^2^.

By inserting a mobile polyethylene layer between the prosthetic femoral head and the acetabular shell to form an additional bearing surface, a DM cup incorporates both Charnley's low-friction concept and the McKee–Farrar concept of increased femoral head-to-neck ratio for maximum stability. However, the widespread use of DM cups has been limited due to the inherent complication of intraprosthetic dissociation (IPD) and the nature of dual articulation, which can accelerate wear of the polyethylene acetabular liner^3–5^. The vitamin E-infused highly cross-linked polyethylene (VEPE) created using vitamin E and its analogs was made available in 2010^6^. VEPE is theoretically known to prevent failure due to oxidative degradation in the body and is resistant to wear; hence, long-term success of DM THA is expected. However, only few studies have directly compared and analyzed the results of DM THA using VEPE with that of fixed bearing (FB) THA in patients at a high risk of dislocation.

Therefore, we aimed to compare the radiologic and clinical results of DM and FB THA in patients at a high risk of dislocation after THA. The primary outcome analyzed was the incidence of postoperative dislocation, and the secondary outcomes were other complications and reoperation. We expected that DM THA would not only show better clinical and radiologic results than that of FB THA but also successfully prevent dislocation after THA.

## Materials and methods

**Figure 1:**
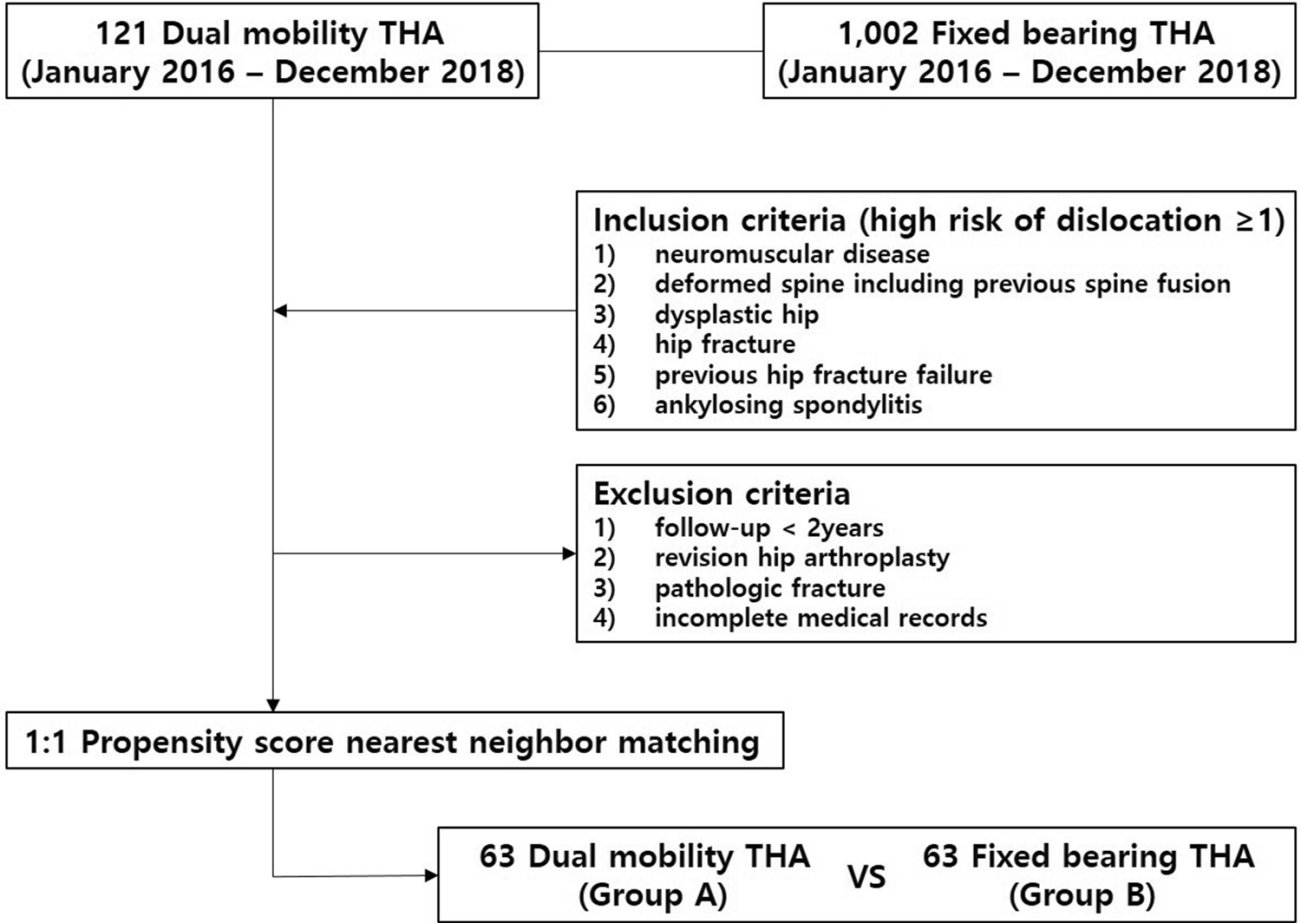
Study design flowchart.

This single-center, retrospective, comparative cohort study enrolled patients who underwent primary THA using a DM cup. From January 2016 to December 2018, 121 DM THA procedures were conducted at our tertiary university hospital. The inclusion criterion of this study was the presence of more than one of the following risk factors for dislocation after THA: neuromuscular disease; deformed spine, including previous spine fusion; dysplastic hip; hip fracture; previous hip fracture failure; and ankylosing spondylitis. Of the 121 patients, 7 patients, who were lost to follow-up, were excluded. Furthermore, 6 patients were excluded because of insufficient follow-up period (< 2 years) or incomplete medical records, and 45 patients were excluded because DM cups were used during revision surgery. Finally, 63 patients with a minimum follow-up of 2 years were included DM group. For selecting the control group participants, the aforementioned inclusion and exclusion criteria were applied to 1,002 patients who underwent FB THA during the same observation period. Fixed 1:1 propensity score matching was performed to minimize any bias that would affect the outcome analysis ^7^. Nearest-neighbor matching was performed considering age, sex, and risk factors for dislocation. After checking the histogram support, 63 FB THA matched patients were selected as the final control group (FB group) (Fig. 1). Each group comprised of 63 THA cases (Table 1). There were no differences between the groups with respect to age, sex, body mass index, cause of THA, risk factor for dislocation, underlying disease, and the American Society of Anesthesiologists status. The mean follow-up period was 3.1 years and 3.5 years in the DM and FB groups, respectively.

**Table 1:** Preoperative demographics in a matched group with at least 2 years average follow-up.

Demographics	DM THA	FB THA	*P* Value
Number	63	63	
Age, mean ± SD, years	61.6 ± 15.0	60.5 ± 13.6	0.667
Gender, F:M	40:23	38:25	0.714
BMI, mean ± SD, kg/m^2^	23.8 ± 4.1	24.5 ± 3.3	0.284
BMD, mean ± SD, T-score	−1.8 ± 1.3	−1.2 ± 1.3	0.019
Follow-up, mean ± SD, years	3.1 ± 0.7	3.5 ± 1.2	1.000
**Cause of THA**			
Osteoarthritis	28	37	0.109
Osteonecrosis	17	10	0.129
Femoral neck fracture	8	5	0.380
Acetabular fracture	1	2	0.559
Internal fixation failure	6	7	0.770
Ankylosed hip	3	2	0.648
**Risk factor of dislocation**			
Deformed spine disease	18	13	0.301
Dysplastic hip	17	21	0.437
Legg-Calvé-Perthes	3	2	0.648
Neuromuscular disorder	12	11	0.818
Hip fracture	7	7	1.000
Previous femoral neck fracture	7	7	1.000
Previous intertrochanteric fracture	9	7	0.593
Previous acetabular fracture	3	3	1.000
Ankylosing spondylitis	4	3	0.697
**Underlying disease**			
Hypertension	27	23	0.466
Diabetes	11	9	0.626
Dyslipidemia	15	3	0.002
Cerebral infarction	8	4	0.225
Chronic kidney disease	2	1	0.559
Hepatitis	2	2	1.000
Cardiac disease	7	2	0.084
Pulmonary disease	3	3	1.000
Hypothyroidism	1	3	0.310
Other neuromuscular disorders	6	4	0.510
Rheumatoid arthritis	3	6	0.299
Ankylosing spondylitis	4	3	0.697
Organ cancer	5	2	0.243
Dementia	1	1	1.000
**American Society of Anesthesiologists status**			
1	17	23	0.251
2	40	33	0.207
3	6	7	0.770

*BMD* bone mineral density, *BMI* body mass index, *DM* dual mobility, *F* female, *FB* fixed bearing, *M* male, *SD* standard deviation, *THA* total hip arthroplasty.

All operations were performed by an experienced arthroplasty surgeon using a posterolateral approach with the patients in the lateral decubitus position. Cementless acetabular and femoral components were used in all patients, and the stem design was determined according to the patient's preoperative template and proximal femoral geometry during surgery (Table 2). Two patients in each group were subjected to spinal anesthesia, while the others were subjected to general anesthesia. In the DM group, the G7 acetabular system (Zimmer Biomet, Inc., Warsaw, IN) was used in all cases. The G7 DM acetabular system device includes a VEPE outer head, which features a third-generation highly cross-linked PE (HXLPE), and a delta ceramic inner femoral head. Titanium alloy acetabular cups are three-dimensional porous cups with a mean pore size of 475 µm, 70% porosity, and coefficient of friction of 1.25. A multi-hole design for acetabular cups was used in this study. In all cases, a 28-mm femoral head was used, and the mean size of the acetabular cup was 53.1 mm. In the FB group, the Trilogy cementless acetabular cup (Zimmer Biomet) and second-generation HXLPE combination was used in 23 cases, whereas the G7 acetabular cup and VEPE combination was used in 40 cases. An elevated HXLPE liner was used in 7 patients who underwent the procedure with a Trilogy cup. Delta ceramic femoral heads were used in all cases, with 32-mm heads used most commonly (n = 38), followed by 36-mm heads (n = 20) and 28-mm heads (n = 5). The mean size of the acetabular cup used in the FB group was 51.8 mm, which was not different from that of the DM group. Postoperatively, all patients were prescribed subcutaneous, low molecular weight heparin for thromboprophylaxis. On the second postoperative day, the patients were instructed to walk with partial weight-bearing with the aid of crutches or a walker, followed by full weight-bearing as tolerated.

**Table 2:** Operative data in DM and FB THA cohort.

Demographics	DM THA	FB THA	*P* Value
**Acetabular component (Zimmer, Biomet, Warsay, IN)**			
Trilogy®	0	23	
G7®	63	40	
**Acetabular liner (Zimmer Biomet)**			
2nd generation HXLPE (Longevity®) standard	0	16	
2nd generation HXLPE (Longevity®) elevated	0	7	
3rd generation HXLPE (E1®)	63	40	
Cup anteversion, mean ± SD, °	21.0 ± 4.5	23.0 ± 4.4	0.021
Cup inclination, mean ± SD, °	40.2 ± 4.3	41.9 ± 4.3	0.056
Cup size, mean ± SD, mm	53.1 ± 3.7	51.8 ± 3.2	0.059
**Prosthetic femoral head**			
Ceramic (Biolox delta, CeramTec)	63	63	
Size, mean ± SD, mm	28 ± 0.0	33.0 ± 2.4	0.001
28 mm	63	5	
32 mm	0	38	
36 mm	0	20	
Neck length, mean ± SD, mm	−0.1 ± 2.6	−0.4 ± 2.3	0.051
**Femoral component (Zimmer, Biomet)**			
Versys® Fiber Metal Taper	7	20	
Wager SL Revision®	7	7	
Microplasty®	49	36	
**Anesthesia**			
General	61	61	
Spinal	2	2	
Operating time, mean ± SD, minutes	77.9 ± 24.2	83.7 ± 37.4	0.311

*DM* dual mobility, *FB* fixed bearing, *HXLPE* highly cross-linked polyethylene, *SD* standard deviation, *THA* total hip arthroplasty.

PolyWare Rev. 7 (Draftware Developers Inc. Vevay, IN, USA) was used to measure the anteversion and inclination of the acetabular cup, and the operating times of the two groups were noted and compared. A postoperative radiologic review was performed at 6 weeks, 3 months, 6 months, and 12 months, and annually thereafter. Standard radiographs with additional Judet views were used to detect periprosthetic osteolysis. Radiolucent lesions ≥ 2 mm around the prosthetic components that were not present immediately postoperatively denoted osteolysis^8^. Changes in inclination > 5° and vertical or ≥ 2 mm horizontal migration of the acetabular component were defined as acetabular component loosening. The medical records and radiographs of patients were analyzed to determine reoperation and presence of postoperative complications such as dislocation, IPD, periprosthetic fracture, venous thromboembolism, and other medical complications. The modified Harris hip score (mHHS) was used to assess the patient-reported outcomes (PROM).

### Statistical analysis

The summary data are expressed as means ± standard deviations for continuous variables and as numbers and frequencies (%) for categorical variables. Continuous variables with a non-normal distribution were analyzed using a Mann Whitney U-test, whereas those with a normal distribution were analyzed using independent t-tests. Categorical data were statistically analyzed using a chi-square test or Fisher’s exact test (n < 40 or t < 1). Propensity scores were calculated using logistic regression analysis. Statistical analysis was performed using Statistical Product and Service Solutions software (version 20.0; SPSS Inc., Chicago, IL, USA), and *p*-values < 0.05 were considered statistically significant.

### Ethics approval and consent to participate

This study followed the World Medical Association Declaration of Helsinki and strengthening the reporting of observational studies in epidemiology (STROBE) guidelines for cohort studies. All procedures performed in studies involving human participants were in accordance with ethical standards, patient information was reviewed by the university human subjects committee and informed consent exemption was obtained from the IRB of our affiliated institutions (Pusan National University Yangsan Hospital, Approval No. 05-2021-032). All experimental protocols were approved by our institutional committee (Pusan National University Yangsan Hospital, Approval No. 05-2021-032).

## Results

At the time of operation, the mean age of the participants in the DM and FB groups was 61.6 and 60.5 years, respectively, and there was no difference between the groups in terms of preoperative demographics, except for the bone mineral density. In both groups, osteoarthritis and osteonecrosis were the most common causes of THA, and deformed spine disease and dysplastic hip were the most common risk factors for postoperative dislocation (Table 1). The mean acetabular cup anteversion of the DM group was significantly smaller than that of the FB group (21.0° vs. 23.0°, *p* = 0.021). The acetabular cup inclination did not differ significantly between the two groups. The mean operation time was 77.9 min and 83.7 min in the DM group and FB group, respectively, without a statistically significant difference (Table 2).

Periacetabular osteolysis was observed in 1 case (1.6%) in the DM group and 2 cases (3.2%) in the FB group, and with no difference between the two groups (Table 3). There was no evidence of implant loosening in either group. Moreover, no reoperation was performed in either group until the final follow-up. Postoperative dislocation occurred in 4 cases (6.3%) in the FB group, but no dislocation was reported after closed reduction. There were no cases of dislocation in the DM group, showing a statistically significant difference compared to the FB group (*p* = 0.042). Intraoperative periprosthetic femoral fracture was observed in 3 cases (4.8%) in the DM group, and intraoperative cerclage wiring was treated without additional complications. Postoperative venous thromboembolism and deep joint infection occurred in 1 case (1.6%) in the FB group, but the difference was not statistically significant. Medical complications were noted in 5 cases (7.9%) in each group, but no serious problems leading to death were observed; there was no statistical difference between the two groups. At the last follow-up, the mHHS showed satisfactory PROM in both groups (DM group, 90.5; FB group, 88.1), with no statistical difference between them.

**Table 3:** Postoperative outcomes in DM and FB THA cohort.

Demographics	DM THA	FB THA	*P* Value
**Radiologic outcome at the last FU**			
Osteolysis (%)	1 (1.6%)	2 (3.2%)	0.559
Implant loosening (%)	0 (0.0%)	0 (0%)	1.000
Reoperation (%)	0 (0.0%)	0 (0.0%)	1.000
**Complications**			
Dislocation (%)	0 (0.0%)	4 (6.3%)	0.042
Intraprosthetic dissociation (%)	0 (0.0%)	0 (0.0%)	1.000
Intraoperative periprosthetic fracture (%)	3 (4.8%)	0 (0.0%)	0.079
Venous thromboembolism (%)	0 (0.0%)	1 (1.6%)	0.315
Deep joint Infection (%)	0 (0.0%)	1 (1.6%)	0.315
Medical complication (%)	5 (7.9%)	5 (7.9%)	1.000
Pneumonia	3	1	0.310
Urinary tract infection	1	1	1.000
Enteritis with Ileus	1	2	0.559
Rheumatoid flare	0	1	0.315
mHHS at the last FU, mean ± SD (range)	90.5 ± 9.8 (68–100)	88.1 ± 9.5 (58–100)	0.245

*DM* dual mobility, *FB* fixed bearing, *FU* follow-up, *mHHS* modified Harris hip score, *THA* total hip arthroplasty.

## Discussion

In this retrospective cohort study, satisfactory clinical and radiologic results were confirmed for DM THA during a follow-up of at least 2 years. Particularly, postoperative dislocation was not observed with DM THA, although all THA procedures were performed using a posterolateral approach and the anteversion of the DM group was smaller than that of the FB group. Thus, this study supported the hypothesis that DM THA is a good option for the prevention of postoperative dislocation in patients at a high risk of dislocation after THA. The strengths of this study included the direct comparative analysis of follow-up data from patients at a high risk of dislocation and its design, in which prostheses from a single manufacturer were used in consecutive patients by the same surgeon.

Among the risk factors associated with postoperative dislocation after THA, the most difficult to predict are the patient-related factors. Neuromuscular disorder, muscle weakness, dysplastic hip, abnormal spinopelvic movement, previous hip fractures, and osteonecrosis of femoral head are well-known patient-related risk factors for dislocation^9–12^. DM cups have been used to preclude dislocations in patients at risk, and various studies have elicited promising results. The risk of dislocation following THA in osteonecrosis of femoral head compared to THA for primary osteoarthritis is higher. Assi C et al. reported that the new generation of DM cup in patients with osteonecrosis of femoral head showed excellent functional early results with no major complication such as dislocation^12^. THA for femoral neck fractures is often associated with a high risk of dislocation secondary to a combination of muscular insufficiency and a propensity for recurrent falls. Tarasevicius et al. described a statistically significant reduction in the dislocation rate with THA using DM as compared to THA with FB (0% vs. 10.4%) during the first postoperative year^13^. Assi CC et al. also reported similar results that the use of DM cup could significantly reduce the rate of dislocation in such a high risk population of patients with femoral neck fracture, and consequently the rate of THA revision surgery and the health cost^14^. Furthermore, DM cups may represent an excellent option in salvage THA performed for failed fixation of hip fractures, which is associated with a high rate of postoperative instability^15^. Many factors, including structural damage after removal of internal fixation and loss of bony landmarks due to trochanteric displacement, are likely to contribute to this instability. In a consecutive series of 1000 patients, Esposito et al. demonstrated that fixed spinopelvic alignment from standing to sitting caused a statistically significant increase in rate of dislocation after THA, with 92% of the patients with dislocation suffering lumbar multilevel degenerative disc disease or surgical spine fusion^16^. Therefore, such patients may benefit from DM THA in reducing postoperative dislocation risk. DM THA has demonstrated excellent mid-term results in patients with neurological diseases or cognitive impairment. The study by Bassiony et al. did not report any case of dislocation of the prosthesis used in hip fractures in patients with Parkinson’s disease^14^. However, most of these studies were limited to specific diseases or were case series without comparison with a control group. In contrast, our study matched and compared not only age and sex but also various risk factors for dislocation between the two groups, in order to exclude confounding factors as much as possible. Several studies have compared the results of DM and FB THAs in general patients, but only the verification of the degree of non-inferiority of DM THA was possible.

It is known that postoperative dislocation usually occurs within 3 months after THA, and joint laxity related to polyethylene wear is the cause of chronic dislocation. The modern DM cup has evolved considerably since the first-generation model of Bousquet in 1974. The retrieval study of polyethylene DM components by D’Apuzzo et al. showed that motion occurs at both articulations, but the motion of the femoral head relative to the inner aspect of the polyethylene head dominant, which produces more wear^17^. Previous studies have reported decreased dislocation rates with primary THA in patients at risk, but with an elevated risk in revision surgery compared to conventional implants. This might result in the release of polyethylene microparticles from the liner and eventually lead to aseptic loosening^13^. Polyethylene wear in the DM system affects the intraprosthetic stability. Excessive eccentricity wear of the inner bearing can lead to loss of constraint of the prosthetic femoral head within the large-diameter polyethylene liner, thus resulting in IPD. The retrieval study of 93 cases with DM system by Neri et al. demonstrated that IPD is a wear-related complication due to contact between the retaining polyethylene rim and the femoral neck^18^. Consequently, biomaterial advancements have replaced first-generation polyethylene with HXLPE to minimize wear due to contact with the femoral neck. Laboratory data illustrates the favorable rate of wear in the contemporary DM cups when compared to that of first-generation implants^19,20^. The DM systems utilized in this series contains VEPE. VEPE is created by adding a free radical scavenger, vitamin E, to polyethylene during processing; vitamin E adequately quenches free radicals that remain after irradiation, eliminating the need for a post-irradiation heating step. Although this study did not seek to assess polyethylene wear, and the follow-up was insufficient to determine this accurately, none of the cases required reoperation for polyethylene wear or IPD. Third-generation HXLPE, such as VEPE, is considered as the most suitable polyethylene material for DM THA in terms of wear and other properties, and it can be expected to prevent dislocation in the mid to long term period.

All 4 cases of postoperative dislocation occurred in the FB group. As for the prosthetic femoral head used for FB, a prosthetic femoral head of 32 mm or larger was used in 58 cases (92%) except 5 cases using 28 mm. Although it is known that the risk of dislocation can be reduced when a head of 32 mm or larger is used compared to a 28 mm or smaller head, the occurrence of dislocation was significantly higher in the FB group than in the DM group. In other words, it can be estimated that the DM cup is an excellent implant for preventing dislocation regardless of the prosthetic femoral head size when THA is performed in patients at high risk of dislocation.

This study had some limitations. First, this was a single-center, retrospective, cohort study, despite accounting for all postoperative radiologic outcomes in our consecutive patients. Second, proper survival analyses could not been performed because of the small sample size; however, to overcome this limitation, a comparative study using 1:1 propensity score matching was conducted to improve the research design. Third, although deformed spine disease and dysplastic hip were the most common risk factors for postoperative dislocation in this study, DM cup was need for various diseases. However, because the number of disease groups was not large, analysis by disease was not performed in this study. Also, it is an obvious limitation that the patients at a risk of dislocation in this study did not include all known dislocation-risk patients. Finally, although the evaluation of the postoperative dislocation during the 2–4 years of follow-up was meaningful, this period was relatively short; hence, the long-term success and polyethylene wear in cases of DM THA using VEPE could not be evaluated. These limitations are obvious obstacles in the generalization of our results, and further multicenter prospective studies are needed to verify their authenticity. We will continue to conduct further follow-up in these patients.

In patients at a risk of dislocation after primary THA, DM cups showed more promising outcomes than did FB. This study reported no dislocation or IPD in patients who underwent primary THA using a DM system at a mean follow-up of 3.1 years, indicating that DM cups could offer the desired early hip stability. Furthermore, DM cups provided good functional results. Contemporary DM bearing with VEPE may be beneficial for patients with a high life expectancy and early compelling hip stability. Based on our findings, we recommend the use of DM cups in all patients at a high risk of dislocation.

## Data Availability

The data utilized are accessible from the corresponding author upon reasonable request.
